# Evidence and knowledge gaps on the disease burden in sexual and gender minorities: a review of systematic reviews

**DOI:** 10.1186/s12939-016-0304-1

**Published:** 2016-01-22

**Authors:** Karel Blondeel, Lale Say, Doris Chou, Igor Toskin, Rajat Khosla, Elisa Scolaro, Marleen Temmerman

**Affiliations:** Ghent University, Ghent, Belgium; Department of Reproductive Health and Research, World Health Organization, Geneva, Switzerland; I.M. Sechenov First Moscow State Medical University, Moscow, Russian Federation

**Keywords:** Sexual and gender minorities, Disease burden, LGBT, Health

## Abstract

Sexual and gender minorities (SGM) include individuals with a wide range of sexual orientations, physical characteristics, and gender identities and expressions. Data suggest that people in this group face a significant and poorly understood set of additional health risks and bear a higher burden of some diseases compared to the general population. A large amount of data is available on HIV/AIDS, but far less on other health problems. In this review we aimed to synthesize the knowledge on the burden of communicable and non-communicable diseases, mental health conditions and violence experienced by SGM, based on available systematic reviews. We conducted a global review of systematic reviews, including searching the Cochrane and the Campbell Collaboration libraries, as well as PubMed, using a range of search terms describing the populations of interest, without time or language restrictions. Google Scholar was also scanned for unpublished literature, and references of all selected reviews were checked to identify further relevant articles. We found 30 systematic reviews, all originally written in English. Nine reviews provided data on HIV, 12 on other sexually transmitted infections (STIs), 4 on cancer, 4 on violence and 3 on mental health and substance use. A quantitative meta-analysis was not possible. The findings are presented in a narrative format. Our review primarily showed that there is a high burden of disease for certain subpopulations of SGM in HIV, STIs, STI-related cancers and mental health conditions, and that they also face high rates of violence. Secondly, our review revealed many knowledge gaps. Those gaps partly stem from a lack of original research, but there is an equally urgent need to conduct systematic and literature reviews to assess what we already know on the disease burden in SGM. Additional reviews are needed on the non-biological factors that could contribute to the higher disease burden. In addition, to provide universal access to health-care for all, more information is needed on the barriers that SGM face in accessing health services, including the attitudes of health-care providers. Understanding these barriers and the additional health risks they impose is crucial to improving the health status of SGM.

**Electronic supplementary material:**

The online version of this article (doi:10.1186/s12939-016-0304-1) contains supplementary material, which is available to authorized users.

## Review

### Background

The Constitution of the World Health Organization (WHO) states that the “enjoyment of the highest attainable standard of health is one of the fundamental rights of every human being without distinction of race, religion, political belief, economic or social condition” [1]. Although medical ethics require health services to be inclusive of all people, some populations have more difficulties than others in reaching this standard of health [2]. Those populations can be defined by age, gender, race or ethnicity, geography, wealth, disability, and also by sexual orientation and gender identity.

Populations defined by sexual orientation and gender identity include individuals with a wide range of sexual orientations, physical characteristics (as in intersex persons), gender identities and expressions, all of which they may experience differently depending on societal gender norms and cultural traditions. For the purposes of this article we have grouped men who have sex with men (MSM), women who have sex with women (WSW), transgender and intersex persons, all defined by the Joint United Nations Programme on HIV and AIDS (UNAIDS), together under the name of ‘sexual and gender minorities’ or SGM [3]. For research, health policy and advocacy reasons, estimating the size of populations of interest is important [4]. Due to stigma associated with homosexuality and non-gender-normative behaviours, estimates of SGM populations are scarce, but studies show that SGM comprise substantial proportions of the population in countries worldwide [5–7].

Data suggest that SGM face a significant and poorly understood set of additional health risks and bear a higher burden of some diseases compared to the general population [8, 9]. There have been international calls to answer the unique health-care needs of SGM [10, 11]. It is therefore indispensable to understand where we are with the knowledge on the health burden of SGM. In 2002 Boehmer showed that research was limited in scope, concentrating mostly on HIV and STIs and MSM [12]. In high-income countries the research scope has been broadening since, but the situation in middle- and low-income countries is less clear [13]. In this review we aimed to collate the knowledge and identify the knowledge gaps on the burden of communicable and non-communicable diseases, mental health conditions and violence experienced by SGM globally, based on available systematic reviews.

### Methods

A review of systematic reviews can aim to bring together a series of reviews on an important topic or summarize reviews on a variety of topics with a common main theme [14]. This review is of the latter type and while it is unlikely to be of interest to clinicians and patients deciding how best to address a specific problem, it may be relevant to policy makers or to addressing questions that cut across the different reviews [15]. The outcome of a review of reviews is typically a summary of review results. Conducting additional meta-analysis in a review of systematic reviews is a major challenge due to the heterogeneity of the methods, analyses and results of the original reviews. Therefore meta-analysis is not a relevant method to this review of systematic reviews and is descriptive in nature.

#### Search strategy

We conducted a review of systematic reviews in concordance with the PRISMA statement [16]. The Cochrane Database of Systematic Reviews and the Campbell Collaboration Library of Systematic Reviews were searched using the following terms to identify reviews on the populations of interest: ‘gay’, ‘lesbian’, ‘transgender’, ‘WSW’, ‘women who have sex with women’, ‘MSM’, ‘men who have sex with men’, ‘same sex’, ‘sexual orientation’, ‘gender identity’, ‘sexual minority’, ‘intersex’ and ‘homosexuality’. We searched PubMed for the terms ‘systematic’ AND ‘review’ combined with a search strategy (see Additional file 1) for sexual and gender minorities (SGM). No language, time or geographical restrictions were applied. The concept of ‘burden of disease’ was not specified in search strategies. Google Scholar was searched in English and references of the included reviews were checked to identify further relevant articles.

#### Inclusion and exclusion criteria

**Fig. 1:**
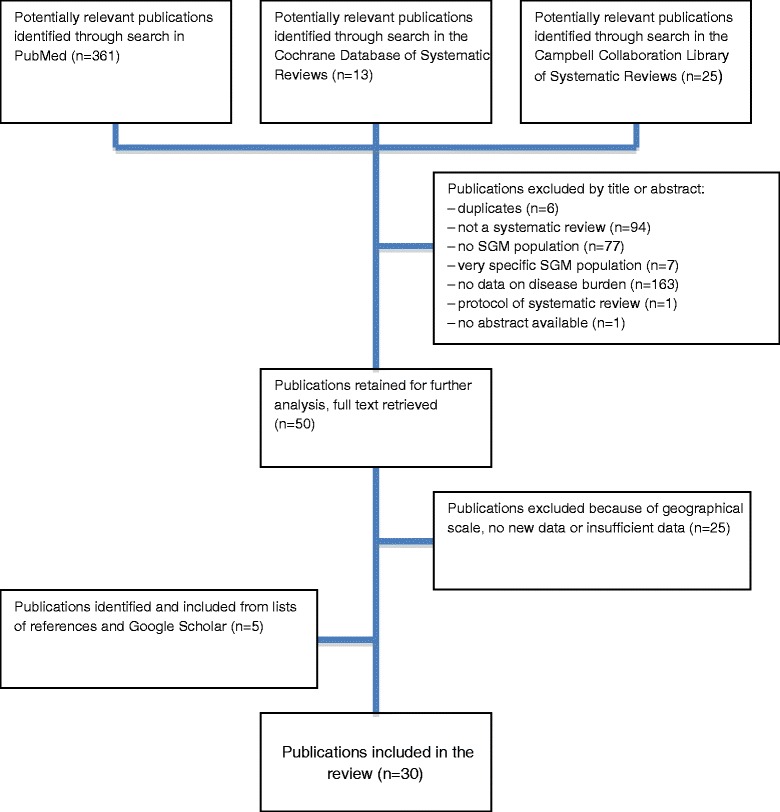
Flowchart of inclusion criteria for reviews

Figure 1 shows how publications were selected for review. Our initial search generated 399 publications. Based on a review of the abstracts, the following exclusions were made: duplicates, one publication lacking an abstract, one protocol of a systematic review, publications that were not systematic reviews, publications without data on disease burden, and publications without data on SGM or with data on only a very specific subset of SGM. We then retrieved and read the full text of 50 retained publications. Those were all publications we found on PubMed, the search on the Cochrane Database of Systematic Reviews and the Campbell Collaboration Library of Systematic Reviews did not generate reviews of interest. Next, we further excluded national reviews if they were included in larger reviews, and reviews that were redundant due to newer published reviews on the same subject and population. Twenty-five articles were retained. An additional five eligible reviews were identified from references and Google Scholar, bringing the total to 30.

#### Quality assessment

The quality of the systematic reviews was assessed with the AMSTAR scale (see Additional file 2) (http://amstar.ca/). AMSTAR stands for A Measurement Tool to Assess Systematic Reviews and was initially developed to assess systematic reviews of randomised controlled trials [17]. However, it is increasingly being used to assess those that include observational studies as well. The scale consists of 11 items. We considered systematic reviews with a score from 0 to 3 as low quality, from 4 to 6 as medium quality, and from 7 up as high quality. The AMSTAR score was not used as an exclusion criterium, but used as an analytic tool. Regardless of the quality, we did not find inconsistencies in the conclusions of the included individual reviews.

#### Data extraction

We created a master table in Microsoft Excel containing key information from each of the included reviews (see Additional file 2): health topic, time range of the search, year published, geographical scope, databases searched, the SGM subpopulation, number of studies included, measures, comparison groups, a summary of the quantitative and/or qualitative data on disease burden and quality assessment score.

### Results

All 30 reviews were originally written in English. Fifteen reviews were global in scope, while 11 were regional and 5 were national. Nineteen reviews were exclusively or predominantly based on data from high-income countries. Nine reviews provided data on HIV [6, 18–25], 12 on other sexually transmitted infections (STIs) [21, 26–36], 4 on violence [37–40], 4 on cancer [32, 41–43] and 3 on mental health and substance use [44–46]. One review provided data on both HIV and STIs [21]; another yielded data on STIs as well as cancer [32]. Twenty-five reviews provided data on MSM, 8 on WSW and 5 on transgender persons (some reviews included more than one subpopulation). Twenty-four studies compared data to the general population or other groups, of which one half on the basis of meta-analysis and the other half based on qualitative observations. Fourteen reviews were considered high quality [6, 19, 20, 22, 23, 26, 27, 32, 36, 38, 42, 44–47], 9 medium quality [18, 24, 30, 31, 34, 35, 37, 39, 40] and 7 low quality [21, 25, 28, 29, 33, 41, 43]. We found no reviews on intersex people. Detailed results are presented in narrative form under five health topic sub-sections, by SGM subpopulation, as shown in Table 1.

**Table 1:** Number of reviews selected, by health topic and subpopulation

Health topic	Men who have sex with men (MSM)	Women who have sex with women (WSW)	Transgender persons	Total number of reviews
HIV/AIDS	6	1	2	9
STI	10	2	1	12
Cancer	3	1	0	4
Mental health and substance use	2	2	1	3
Violence	4	2	0	4
*Total*	*25*	*8*	*5*	*30*

#### HIV/AIDS

Nine systematic reviews assessed the burden of HIV in SGM, including 5 global and 4 regional reviews [6, 18–25]. Six of these reviews were on MSM, 2 were on male-to-female transgender persons and 1 was on WSW.

##### Men who have sex with men (MSM)

Among the 6 reviews that provided data on MSM, 3 were exclusively on MSM. One review was global [24] and 5 were regional [6, 18, 19, 23, 25], of which 4 were mainly on low- and middle-income countries (LMICs). Except for one review from 2007, all were published in 2010 or later. All reviews showed a high burden of HIV in MSM. The region with the highest HIV prevalence in MSM was the Caribbean with 25.4 %, followed by sub-Saharan Africa with 17.9 % [24]. In contrast, some countries in the Middle East and North Africa show very low HIV prevalence in MSM [6]. In Asia, high rates of incident HIV-infections in MSM were observed wherever studied [18], and across the region of Latin America and the Caribbean MSM was the population most affected by HIV [19]. MSM in LMICs have on average a 19.3 times higher chance of being infected with HIV compared with the general population [23]. In high-income countries, Sullivan et al. showed that among new HIV cases, the proportion attributed to male–male sex increased in all the included countries between 2006 and 2011 [25].

##### Transgender persons

Two systematic reviews, both of high quality, provided data on HIV infection in male-to-female transgender persons [20, 22]. The reviews were global, collectively including studies from 20 countries. One publication calculated an HIV prevalence of 14.7 % in transgender persons who did not engage in sex work and 27.3 % in those who did [20]. The second review estimated a prevalence of 19.1 % and reported that this population has a 48.8 times higher chance of being infected with HIV than the general population of adults aged between 15 and 49 [22]. We found no reviews on HIV infection in female-to-male transgender persons.

##### Women who have sex with women (WSW)

A low quality systematic review on STIs in WSW globally did not generate any HIV prevalence data in this population [21].

#### Sexually transmitted infections

Twelve systematic reviews included data on STIs other than HIV in SGM, including 6 global, 4 regional and 2 national-level reviews [21, 26–36]. Nine of these reviews were on MSM, 2 on WSW and 1 on MSM and transgender persons.

##### MSM

Three reviews reported on syphilis in MSM. Zoni et al. showed high rates of active syphilis prevalence in MSM in Latin America, with rates of 7.5 % or more in half of the included studies [27]. In China, the median syphilis prevalence, based on a review of four studies in MSM, was 14.5 %, making MSM the group at highest risk for syphilis in China [28]. Caceres et al. found that syphilis prevalence among MSM in 16 LMICs ranged from 1.5 % in Bangladesh to 29 % in Peru [35].

Hepatitis prevalence was reported in four reviews. A 2003 low quality global systematic review on hepatitis A virus (HAV) did not find an important difference in anti-HAV positivity between MSM and control groups [29]. Two reviews on hepatitis B and C viruses (HBV and HCV) in Europe presented disaggregated data for MSM [30, 31]. One showed higher hepatitis C virus antibodies and hepatitis B surface antigen prevalence than in the general population, based on three country estimates [30]. In LMICs, data on HBV prevalence was even sparser. With rates of HBV up to 38 % in Argentina and 31 % in Vietnam, MSM seem to be a high-risk group [35]. A global review comparing incidence of acute HCV in HIV-negative versus HIV-positive MSM found that the incidence in the latter group was four times higher; the incidence rates in HIV-negative MSM were similar to heterosexual populations [36]. The Pan American Health Organization (PAHO) is currently conducting a review of hepatitis B and C in the countries of the Americas; so far the findings point to a higher burden for MSM (personal communication, Rafael Mazin, focal point for HIV/STIs and hepatitis in key populations, PAHO, 30 May 2014).

In one low quality global review, prevalence of herpes simplex virus 2 (HSV-2) was demonstrated to be higher among male homosexual than among male heterosexual populations [33].

*Chlamydia trachomatis* and *Neisseria gonorrhoeae* have not been the subject of global systematic reviews in SGM. One review reported data from nine LMICs with prevalence rates ranging from 0 % (Peru) and 2.1 % (Nepal) up to 14.9 % and 16.1 % (both in Timor-Leste) [35]. A national review on *C. trachomatis* in Australia designated MSM amongst the populations with the greatest burden, calculating a pooled prevalence for rectal *C. trachomatis* of 5.6 % [34].

In a high quality global systematic review, Machalek et al. showed a pooled prevalence of any type of anal human papillomavirus (HPV) of 53.6 % in HIV-negative MSM and 89.0 % in HIV-positive MSM [32]. High-grade anal intraepithelial neoplasia was present in 20–30 % of all MSM [32].

##### WSW

Two global systematic reviews on bacterial vaginosis and on STIs in WSW showed a positive association between a history of female sexual partners and risk of bacterial vaginosis [21, 26]. There was no evidence of a higher burden of STIs in WSW compared with the general population of adult women [21].

##### Transgender persons

Zoni et al. found syphilis prevalence rates ranging from 6.5 % in El Salvador to 43.3 % in Brazil, the latter being the highest rate among high-risk populations (MSM, sex workers and transgender persons) in Latin America and the Caribbean [27]. Preliminary results of an ongoing review on hepatitis B and C by PAHO in the region of the Americas show a higher burden for male-to-female transgender persons (personal communication, Rafael Mazin, focal point for HIV/STIs and hepatitis in key populations, PAHO, 30 May 2014).

#### Cancer

Four reviews, two high and two low quality, included data on cancer in SGM; all were global in scope [32, 41–43]. Three of these reviews were in MSM and 1 in WSW; no reviews were found on cancer burden in transgender persons.

##### MSM

Anal HPV is common in MSM, as are HBV and human herpesvirus 8 (HHV-8) infections. These viruses can lead to anal cancer, liver cancer and Kaposi sarcoma, respectively. One review considered MSM at high risk for anal cancer, and found that HIV-positive MSM were at highest risk [43]. Systematic reviews on HPV and on cancer in general in MSM found that HIV-positive MSM had a high incidence of anal cancer, similar to the incidence of cervical cancer in the general female population before the introduction of national cervical screening programmes [32, 41]. Boehmer et al. found that only 1 of the 47 studies included in their review on cancer in MSM was not linked to STIs [41].

##### WSW

Meads & Moore systematically reviewed studies on breast cancer in lesbian and bisexual women. This review included nine studies with prevalence estimates, of which two showed higher prevalence of breast cancer in lesbian and bisexual women then in heterosexual women, four showed no difference, one showed mixed results and two could not be compared. Though the review was of high quality, all the included studies were small and of poor quality. The reviewers concluded that there was no convincing evidence of a higher disease burden of breast cancer in WSW [42].

#### Mental health and substance use

Three high quality reviews – 2 global and 1 national – provided data on mental health and substance use in SGM [44–46]. Two of these were in MSM and WSW while 1 was in transgender persons.

##### MSM and WSW

In their global review, King et al. calculated an overall 2.47 times increased risk of lifetime suicide attempts in lesbian, gay and bisexual (LGB) people compared to heterosexuals; the risk ratio for lifetime suicidal ideation was 2.04, and it was 2.05 for 12 months prevalence of depression, one of the most common mental illnesses [45]. The reviewers also calculated a pooled risk ratio of 1.88 in gay and bisexual males for 12 months prevalence of anxiety disorders (specific disorders were not specified), but the results for lesbian and bisexual females were less convincing due to heterogeneity in the study methods (I2 = 49.2 %). The review also found that alcohol and substance dependence were at least 1.5 times more common in these populations than in the heterosexual population. Marshal et al. calculated that the odds for substance use among LGB youth globally were 190 % higher than for heterosexual youth [46].

##### Transgender persons

We found data on mental health in transgender persons in a systematic review on the prevalence of HIV and contextual factors potentially associated with HIV risk in the United States of America (USA). High rates of suicidal thoughts (weighted mean, 53.8 %) and lifetime suicide attempts (weighted mean, 31.4 %) were reported in male-to-female transgender persons [44].

#### Violence

Violence is one of the more visible health issues in SGM, and the subject of many advocacy efforts. We found four systematic reviews on the prevalence of violence in SGM, including 1 global, 1 regional (North America and Europe) and 2 national-level reviews (USA) [37–40]. All four were on LGB people; there were no reviews on violence in transgender persons.

##### MSM and WSW

A global review found high rates of physical assault (28 %) and sexual assault (27 %) in LGB individuals, and suggested that they experience more victimization than heterosexuals [37]. Peterson et al. concluded that rates of adult sexual assault among gay and bisexual males in North America and Europe far exceed the rates in community samples of men [40]. LGB people in the USA seem to be at increased risk for sexual violence victimization compared with their heterosexual counterparts [38]. Focusing on the subset of intimate partner violence, Finnerman & Stephenson concluded that emergent evidence in the USA demonstrates that prevalence of intimate partner violence is high in male–male partnerships [39].

### Discussion

Our review found that HIV and STIs are the most researched topics for those studying the disease burden in SGM, and that the subpopulation of MSM is the most intensively studied.

The six included reviews on HIV in MSM (all published in 2007 or later) show a high burden in this population. UNAIDS reports that the median HIV prevalence among MSM exceeds 1 % in all regions of the world and is substantially higher than prevalence among men overall, in every context studied [47]. Furthermore, there is no evidence of a decline in the HIV epidemic in MSM [18, 24, 47]. HIV prevalence among MSM in some low-income countries could be underreported because the data are too limited; male same-sex sexual behaviour takes multiple forms and HIV has huge potential to increase further in MSM in the next decade [48, 49]. Abu-raddad et al. indicated growing – in some cases rapidly growing – HIV epidemics in MSM in several countries, including Egypt, Morocco, Pakistan, Sudan, Tunisia and Yemen [49]. Systematic reviews of the existing research of HIV burden in MSM seem to be up to date and of reasonable quality, though original research and data are still lacking in many countries.

Limited research on HIV in male-to-female transgender persons suggests a high burden. For WSW, a review generated no results but was low in quality [21]. For female-to-male transgender persons and intersex people we found no reviews. The feasibility of conducting a systematic review on disease burden of HIV in these populations should be investigated.

Reviews on STIs reported a high burden of syphilis in MSM in Latin America, China and LMICs where prevalence was measured. A report by the European Surveillance of Sexually Transmitted Infections (ESSTI) network confirms that MSM also bear a disproportionate burden of syphilis across Western Europe and that there is clear evidence that it has increased considerably [50]. Syphilis has been identified as a growing problem among MSM in a number of countries, based on the Global AIDS Response Progress Reports [51].

HAV burden was not found to be higher in MSM compared to heterosexual men in high income countries [29], but a more recent report on viral hepatitis in European MSM did show an elevated prevalence [52]. Increasing attention is being paid to HBV as an STI in MSM. While a 2004, non-sysytematic, review on disease burden of HBV only mentioned homosexual activity as one of the risk factors, alongside heterosexual activity [53], a more recent epidemiological report estimated that HBV incidence among MSM is 20 times higher than in the general population [52]. The same report also looked at HCV, but did not clarify whether its high burden was only in HIV-positive MSM or in MSM in general [52].

A 2014 protocol for a global systematic review on the prevalence of HHV-8 described the investigators’ intention to examine the hypothesis that MSM have a high prevalence of the HHV-8 antibody – the infectious disease underlying Kaposi sarcoma – and to determine if the prevalence is increasing [54]. The results have not been published yet.

Our results suggested a high burden of *C. trachomatis* in MSM, based on sparse data from medium quality reviews [34, 35]. Moreover, the most recent annual epidemiological report of the European Centre for Disease Prevention and Control (ECDC), published in 2013, indicated that 99 % of cases of the L2b variant of *C. trachomatis*, which causes the majority of lymphogranuloma venerum infections, are in MSM [55]. Among those cases with known HIV status, 88 % were HIV-positive.

Data from systematic reviews on the burden of *N. gonorrhoeae* in SGM were too scarce to support any conclusions, but an ESTTI report suggested that MSM bear a disproportionate burden of gonococcal infection in many Western European and some Central and Eastern European countries [50]. The ECDC report also indicated that among all *N. gonorrhoeae* cases diagnosed in men in Europe in 2011, 53 % were in MSM [55]. Moreover, there are indications that MSM are one of the core groups that are key to the emergence, onward transmission and outbreaks of antimicrobial-resistant *N. gonorrhoeae* [56].

The burden of anal HPV is well documented in MSM; the review by Machalek at al. showed high prevalence in MSM in general and very high prevalence in HIV-positive MSM in particular [32]. We found no review on the prevalence of oral HPV, though studies show it is high in MSM, and is a risk factor for oropharyngeal cancer [57].

The systematic review on STI/HIV in WSW did not show a higher burden in this population [21]. Nonetheless, the authors concluded that WSW should not be presumed to be at lower risk based on their sexual orientation, since they can acquire HIV and other STIs through other modes, including injection drug use and sexual contact with high-risk male partners.

Available research on cancer burden in MSM focuses mainly on cancers that are causally linked to STIs; most other cancers have yet to be studied in this subpopulation [41]. Lee et al. showed that there might be reason to believe there are also disparities in other cancers, as sexual minorities in the USA are 1.5 to 2.5 times more likely to be smokers than the heterosexual population [58]. Smoking is a major risk factor for lung cancer and a risk factor for a myriad of other cancers.

Mental health is a poorly reviewed health issue in SGM. The most recent global systematic review on mental health in SGM, although high quality, dated from 2008 and suggested an elevated burden in a variety of mental health issues, but it did not include transgender persons [45]. Eating disorders and obesity were not discussed, although they are mentioned in a review on the health of SGM by the United States Institute of Medicine [9]. Regarding substance use, we found that sexual orientation is an important mediating factor of adolescent substance use [46]. Adding to this, Bourne et al. conducted a global literature review on drug use in MSM and concluded that the prevalence of drug use is high [59]. Some party drugs used by MSM, such as crystal methamphetamine or gamma-butyrolactone, can have detrimental effects on mental and sexual health (an internet survey in Asia reported 4 % and 2.3 % use in MSM, respectively, in the last six months) [59].

Lastly our results show that MSM and WSW are confronted with more violence than their heterosexual counterparts. However, most of the studies were from the USA and other high-income countries; we might expect to see even higher rates of violence in SGM in countries where homosexuality is criminalized. A study from the USA on violence in transgender persons assessed self-report surveys and needs assessments, hotline calls, social service records and police reports [60]. All these sources indicated that violence against transgender persons starts early in life, that they are at risk for multiple types and repeated incidents of violence, particularly sexual violence, and that this threat lasts throughout their lives.

Except for the reviews that reported on cancer and mental health, there are no systematic reviews on non-communicable diseases in SGM. Nevertheless, there is a growing body of research on the health of SGM. We analysed data from systematic reviews only; therefore the results of research that have not yet been included in a systematic review are not reflected in this article. More extensive reporting, which includes the results of these studies and of studies on risk, contextual and contributing factors of health disparities and specific health issues in SGM, is beyond the scope of this review. Methodologically, this review is limited by the databases we searched and the use of exclusively English search terms on Google Scholar.

### Conclusions

Our review primarily shows that there is a high burden of disease for certain subpopulations of SGM in HIV, STIs, STI-related cancers and mental health conditions, and that they also face high rates of violence. Secondly, our review revealed many knowledge gaps. Those gaps partly stem from a lack of original research, which needs to be addressed, but there is an equally urgent need to conduct high quality systematic and literature reviews to assess what we already know on the disease burden in SGM.

We recommend that systematic reviews on HIV and HPV in MSM be regularly updated. Reviews on herpes in MSM, STIs in WSW, and on mental health, violence and substance use in MSM and WSW, urgently need to be updated. Special attention should be given to violence based on sexual orientation and gender identity. Global reviews on syphilis, hepatitis, *C. trachomatis* and *N. gonorrhoeae* in MSM, and on all of the most common STIs, mental health and violence in the transgender population need to be conducted. In particular, research on female-to-male transgender persons should be considered, as there are virtually no epidemiologic data on the burden of disease in this population. Literature reviews should include publications on the burden of cancers that are not related to infectious causes and other non-communicable diseases in all SGM. It would facilitate this process if health research, surveys and statistical databases systematically included sexual orientation and gender identity as a demographic characteristic, where possible. There is also a geographical research gap since most of the systematic reviews included studies that were all or mostly from high-income countries. New reviews could capture research from LMICs by broadening and deepening search strategies. The quality of the systematic should also improve, as less than half were of high quality,

The majority of the currently available systematic reviews are on MSM and the burden of HIV and STIs. This suggests that a substantial portion of the burden of disease in SGM is related to their sexual behaviours, which means it is preventable. WHO and UNAIDS have been building on that knowledge, issuing guidelines, recommendations and tools, including tools for estimating the numbers of specific populations, and for monitoring and evaluating policies and interventions to remediate the burden of HIV and STI in these populations [2, 61–63]. Additional reviews should be conducted on the non-biological factors that contribute to higher disease burden. In order to provide universal access to health-care, more knowledge is needed on the barriers that SGM face in accessing health services, including the attitudes of health-care providers, who are ethically bound to provide good quality services, regardless of the personal history or characteristics of the patient. Understanding these barriers and the additional health risks they impose is crucial to improving the health status of SGM.

## Additional files

Additional file 1:
**Search strategy, PubMed, sexual and gender minorities.** (DOCX 105 kb) Additional file 2:
**Summary table of review of systematic reviews.** (XLSX 17 kb)

## Abbreviations

AIDS: acquired immunodeficiency syndrome
ECDC: European Centre for Disease Control and Prevention
ESSTI: The European Surveillance of Sexually Transmitted Infections
HAV: hepatitis A virus
HBV: hepatitis B virus
HCV: hepatitis C virus
HHV-8: human herpesvirus 8
HIV: human immunodeficiency virus
HPV: human papillomavirus
HSV-2: herpes simplex virus 2
LMIC: low- and middle-income country
MSM: men who have sex with men
PAHO: Pan American Health Organization
SGM: sexual and gender minorities
STI: sexually transmitted infection
UNAIDS: The Joint United Nations Programme on HIV and AIDS
WHO: World Health Organization
WSW: women who have sex with women
