# Supplementary material for: Evidence and knowledge gaps on the disease burden in sexual and gender minorities: a review of systematic reviews
Source: Int J Equity Health. 2016 Jan 22;15:16. doi: 10.1186/s12939-016-0304-1 (PMC4724086; doi:10.1186/s12939-016-0304-1)
Supplement: Additional file 1: — Search strategy, PubMed, sexual and gender minorities. (DOCX 105 kb) [file 12939_2016_304_MOESM1_ESM.docx]

# Additional files

**Additional file 1: Search strategy, PubMed, sexual and gender minorities**

homosexuality[Mesh] OR bisexuality[Mesh] OR transsexualism[Mesh] OR "transgendered persons"[Mesh] OR homophobia[Mesh] OR "Health Services for Transgendered Persons"[Mesh] OR homosexuality[TIAB] OR homosexual[TIAB] OR homosexual*[TIAB] OR homo-sexual[TIAB] OR homo-sexual*[TIAB] OR (“same sex”[TIAB] NOT twins) OR (“same sex” AND twins AND homosexuality) OR "non heterosexual"[TIAB] OR "same gender loving"[TIAB] OR "same sex attracted"[TIAB] OR queer*[tiab] OR LBGT[TIAB] OR LBGT*[TIAB] OR LGBT[TIAB] OR LGBT*[TIAB] OR GLBT*[TIAB] OR GLB*[TIAB] OR LGB*[TIAB] OR LGBTQ*[TIAB] OR LGBTI*[TIAB] OR sexual minorit*[tiab] OR gender minorit*[tiab] OR “sexual orientation”[tiab] OR gay[TIAB] OR gays[TIAB] OR (MSM[TIAB] NOT “metal-semiconductor-metal”) OR "men who have sex with men"[TIAB] OR (MSW[TIAB] NOT waste) OR "male sexworkers"[TIAB] OR "male sex workers"[TIAB] OR sissy[TIAB] OR sissies[TIAB] OR lesbian[TIAB] OR lesbian*[TIAB] OR lesbians*[TIAB] OR WSW[TIAB] OR "women who have sex with women"[TIAB] OR tomboy*[TIAB] OR bisexuality[TIAB] OR bisexual*[TIAB] OR bi-sexual*[TIAB] OR transgender*[TIAB] OR trans-gender*[TIAB] OR transvestism[TIAB] OR transvestite[TIAB] OR transsexual*[TIAB] OR transsexualism*[TIAB] OR "trans man"[TIAB] OR "trans men"[TIAB] OR "trans women"[TIAB] OR "trans woman"[TIAB] OR transgendered[TIAB] OR “sex change” [TIAB] OR “sex reassignment surgery”[TIAB] OR cross-dress*[TIAB] OR (intersex AND human) OR (intersex* AND human) OR “gender variant”[TW] OR “gender atypical”[TW] OR “gender identity disorder”[TW] OR transgenderist[TIAB] OR bigender[TIAB] OR “bigender person”[TIAB] OR “drag queens”[TIAB] OR “drag kings”[TIAB] OR “gender queer”[TIAB] OR pansexual[TIAB] OR omnisexual[TIAB] OR “questioning people”[TW] OR “questioning youth”[TW] OR homophob*[TIAB] OR homo-phob*[TIAB] OR transphob*[TIAB] OR trans-phob*[TIAB] OR "anti homosexual bias"[TIAB] OR "anti gay bias"[TIAB] OR “gender dysphoria”[TIAB] OR hijra[TIAB] OR aravanis[TIAB] OR kothi[TIAB] OR “money boys”[TIAB] OR kwandengue[TIAB] OR “male street laborers”[TIAB] OR kathoy[TIAB] OR kathoey[TIAB] OR fafafine[TIAB] OR “sworn virgins”[TIAB] OR two-spirit[TIAB] OR mashoge[TIAB] OR pengkids OR metis[TIAB] OR “mak nyah”[TIAB].
